# HERVs Expression in Autism Spectrum Disorders

**DOI:** 10.1371/journal.pone.0048831

**Published:** 2012-11-14

**Authors:** Emanuela Balestrieri, Carla Arpino, Claudia Matteucci, Roberta Sorrentino, Francesca Pica, Riccardo Alessandrelli, Antonella Coniglio, Paolo Curatolo, Giovanni Rezza, Fabio Macciardi, Enrico Garaci, Simona Gaudi, Paola Sinibaldi-Vallebona

**Affiliations:** 1 Department of Experimental Medicine and Biochemical Sciences, University of Rome “Tor Vergata”, Rome, Italy; 2 Pediatric Neurology Unit, Neuroscience Department, University of Rome “Tor Vergata”, Rome, Italy; 3 Department of Infectious, Parasitic and Immune-Mediated Diseases, Italian National Institute of Health, Rome, Italy; 4 Department of Psychiatry and Human Behavior, University of California Irvine, Irvine, California, United States of America; 5 Department of Psychiatry and the Behavioral Sciences, Keck School of Medicine at University of Southern California, Los Angeles, California, United States of America; 6 Department of Medicine, Surgery and Dentistry, University of Milan, Milan, Italy; King's College London, United Kingdom

## Abstract

**Background:**

Autistic Spectrum Disorder (ASD) is a heterogeneous neurodevelopmental disorder, resulting from complex interactions among genetic, genomic and environmental factors. Here we have studied the expression of Human Endogenous Retroviruses (HERVs), non-coding DNA elements with potential regulatory functions, and have tested their possible implication in autism.

**Methods:**

The presence of retroviral mRNAs from four HERV families (E, H, K and W), widely implicated in complex diseases, was evaluated in peripheral blood mononuclear cells (PBMCs) from ASD patients and healthy controls (HCs) by qualitative RT-PCR. We also analyzed the expression of the *env* sequence from HERV-H, HERV-W and HERV-K families in PBMCs at the time of sampling and after stimulation in culture, in both ASD and HC groups, by quantitative Real-time PCR. Differences between groups were evaluated using statistical methods.

**Results:**

The percentage of HERV-H and HERV-W positive samples was higher among ASD patients compared to HCs, while HERV-K was similarly represented and HERV-E virtually absent in both groups. The quantitative evaluation shows that HERV-H and HERV-W are differentially expressed in the two groups, with HERV-H being more abundantly expressed and, conversely, HERV-W, having lower abundance, in PBMCs from ASDs compared to healthy controls. PMBCs from ASDs also showed an increased potential to up-regulate HERV-H expression upon stimulation in culture, unlike HCs. Furthermore we report a negative correlation between expression levels of HERV-H and age among ASD patients and a statistically significant higher expression in ASD patients with Severe score in Communication and Motor Psychoeducational Profile-3.

**Conclusions:**

Specific HERV families have a distinctive expression profile in ASD patients compared to HCs. We propose that HERV-H expression be explored in larger samples of individuals with autism spectrum in order to determine its utility as a novel biological trait of this complex disorder.

## Introduction

Autism spectrum disorder (ASDs) is a complex neurodevelopmental disorder characterized by varying levels of impairment in social interaction and communication as well as stereotypes and rigid patterns of behaviour [1]. The prevalence rates of ASD have been increasing worldwide, the most recent prevalence studies indicating that they are present in 6 per 1000 children. The ASD etiology is still unknown, but data suggest a likely multi-factorial origin with a strong genetic basis [2]. Twin studies have shown an inheritance of 92% [3]. While the high heritability of ASD is well established, the exact underlying causes and relevant mutations are identified in only a minority of patients. Rare genetic disorders and chromosomal abnormalities are in fact thought to occur in only 10% of ASD cases, approximately.

Possible solutions to the many questions regarding the heritability of complex diseases were eagerly expected after the completion of the human genome sequence. From these studies, novel perspectives emerged, suggesting that protein-encoding genes are not the only actors in the story, that the entire genome is subjected to plasticity and is intimately tied to disease states, and that genes and environmental conditions do indeed interact, thus laying new grounds for ASD etiology [4], [5]. In support to this view, *de novo* and inherited Copy Number Variants (CNVs), and inherited point mutations, have been increasingly found to associate with ASD [6], [7]. Moreover, some newly identified polymorphisms point to non-coding regions and raise the possibility that regulatory in addition to coding variants may contribute to the genetics of autism [8].

Mobile retroelements, which make up almost 50% of the human genome [9], are known to generate extensive structural variations [10], [11] and are regarded as key players in genome function. Among them, Human Endogenous Retroviruses (HERVs) constitute about 8–9% of the human genome (9). They closely resemble infectious retroviruses [12] and are considered to be remnants from ancient germ line viral infections, integrated as provirus in chromosomal DNA. Despite the structural similarities shared with exogenous retroviruses, the vast majority of HERV sequences are in evolutionary equilibrium with the host genomes and their mRNAs are variably expressed in a variety of cell types and tissues [13]–[15]. During evolution, HERVs were amplified and spread throughout the genome by repeated events of retrotransposition and/or reinfection; their integration in any location of the genome can alter in consequence the structure and/or function of other genes [16], [17]. Indeed, HERV activity is implicated in many complex diseases that have multifactorial etiology and genetic basis, including type 1 diabetes [18], various types of tumors [19], [20], autoimmune diseases (for a review [21]) and neuropathogenic diseases such as multiple sclerosis [22].

Variations in specific HERV families have also been detected in schizophrenia. Elevated levels of HERV-W have been detected in peripheral blood mononuclear cells (PBMCs) of patients with recent-onset and chronic schizophrenia [23]. A statistically significant reduction in the expression of gag protein encoded by HERV-W in neurons and astroglial cells has been found in brains from individuals with schizophrenia [24]. Only a subgroup of the HERV-K family (HERV-K10) was significantly overrepresented in brains sample of patients with schizophrenia [25].

To the best of our knowledge, HERV elements have not been assessed in ASD patients as yet. In this study we have investigated the possible association between the presence and expression levels of the four major human HERV families and the onset and disease severity of autism.

## Materials and Methods

### Patients and healthy controls (Participants)

The study included a group of 28 Caucasian children with either idiopathic primary autism or Pervasive Developmental Disorder-Not Otherwise Specified (PDD-NOS). Patients (ASD) were 32 to 113 month-old (median age 58.5 months), of which 22 were males and 6 females. Patients were recruited among those attending the pedopsychiatry outpatient Unit of “Tor Vergata” University Hospital (Rome). All patients had met the DSM-IV-TR diagnosis criteria for autistic disorder and were diagnosed according to Autism Diagnostic Interview-Revised (ADI-R), Autism Diagnostic Observation Schedule (ADOS) and CARS (Child Autism Rating Scale) and their developmental level was assessed by using the Psycho-educational Profile-Third edition (PEP-3). The skills and behaviors were reported as Adequate, or, if altered, as Mild, Moderate and Severe according to degree of impairment in each area of analysis (Table 1).

**Table 1:** Clinical features and assessments obtained using the ADI-R, ADOS, CARS and PEP-3 diagnostic scales.

				ADI				ADOS				CARS	PEP-3		
Patients code	Age (months)	Gender	ASS*	(a)	(b)	(c)	(d)	(a)	(b)	(c)	(d)		C^1^	M^2^	MB^3^
1	51	F	6	15	5	6	4	5	14	4	2	50.5	moderate	moderate	moderate
2	37	M	4	18	9	10	4	4	9	1	1	45.5	moderate	moderate	severe
3	49	M	6	21	11	11	3	7	10	4	5	47.5	severe	severe	severe
4	49	M	4	1	5	7	1	6	7	1	1	41.5	moderate	mild	moderate
5	56	M	3	8	8	10	4	2	8	2	1	47.5	moderate	mild	moderate
6	64	M	6	15	13	10	3	5	7	1	0	41.5	mild	moderate	adequate
7	51	F	7	23	5	11	5	6	14	3	4	49	moderate	moderate	severe
8	74	M	8	27	12	12	4	8	12	1	2	53	moderate	moderate	moderate
9	74	M	5	31	22	5	2	3	11	0	0	33.5	moderate	moderate	moderate
10	32	M	2	19	10	9	3	1	7	3	5	39.5	severe	severe	severe
11	39	M	7	21	14	12	3	8	14	4	5	51.5	severe	severe	severe
12	69	F	6	22	14	9	3	4	13	2	2	45	moderate	mild	moderate
13	69	F	6	20	12	10	3	5	14	2	2	43	moderate	mild	moderate
14	81	M	7	22	12	5	3	8	12	3	2	45	moderate	moderate	moderate
15	45	M	6	21	12	11	3	4	13	4	3	45	severe	severe	severe
16	55	M	6	27	18	12	4	6	7	1	1	28.5	moderate	mild	mild
17	42	M	7	16	13	6	3	8	14	4	5	31.5	adequate	adequate	adequate
18	81	M	6	31	15	13	5	6	10	2	6	51	moderate	moderate	moderate
19	61	M	9	28	13	9	3	8	16	4	5	50	severe	severe	severe
20	42	M	6	14	9	10	3	6	14	4	2	41.5	moderate	mild	moderate
21	60	M	3	18	6	12	4	4	4	0	3	45.5	moderate	mild	moderate
22	112	M	10	27	24	11	1	8	13	2	6	ne	adequate	adequate	adequate
23	113	M	2	ne∧	ne	ne	ne	0	4	0	1	33	adequate	adequate	adequate
24	55	F	6	20	12	3	4	6	13	4	5	ne	severe	mild	moderate
25	80	M	4	ne	ne	ne	ne	4	8	4	3	ne	severe	moderate	moderate
26	60	M	6	9	8	8	2	6	11	1	1	25	adequate	adequate	adequate
27	70	M	3	14	12	6	4	4	4	1	3	ne	moderate	mild	moderate
28	57	F	6	22	11	8	3	7	12	4	3	51	severe	severe	severe

^ ne: not evaluated.

* ASS: Autism Severity Score.

1 : Communication;

2 : Motor;

3 : Maladaptative behaviors.

Patients with known infectious, metabolic or genetic diseases, chromosomal abnormalities, seizures, identifiable neurological syndromes or focal signs were excluded from the study. All children were tested for chromosomal abnormalities and none had fragile X syndrome. All patients were free of drugs at the time of blood collection.

The patient group was compared with a control group of healthy Caucasian children with normal development who attended the outpatient facilities of the “Tor Vergata” University Hospital (Rome) for routine visits. The control group included 28 children (HC), who were matched to the patients by age and gender (Table 2), 32 to 108 month-old (median age 60.0 months), 22 of whom were males and 6 females. None of them had a history of neurological, psychiatric or infectious disorders.

**Table 2:** Demographic information for the ASD and control cohorts.

		*ASD patients* *(n = 28)*	*Healthy controls* *(n = 28)*	*p value*
*Gender*	*Male*	22	22	
	*Female*	6	6	
	*Ratio (M/F)*	3.67	3.67	1
*Median age (range)*		58.5 (32–113)	60 (32–108)	0.873

The University Hospital of “Tor Vergata” Ethics Committee approved of the study and all examinations were performed after receiving written informed consent of the parents.

### Preparation of samples

PBMCs from heparinized blood samples from both ASD and HC groups were analyzed immediately after collection (T_0_) or after stimulation in culture for 72 hours (T_72_) with the T-lymphocyte-specific mitogen phytohemagglutinin (PHA), 2 µg/ml (Sigma, St Louis, MO) and human recombinant interleukin-2 (IL-2), 20 U/ml (Chiron corporation, Emeryville, CA). PBMCs were cultured in RPMI 1640 medium (Life Technologies, Paisley, Scotland, UK) supplemented with 12% fetal calf serum (FCS, Life Technologies), 2 mM glutamine (Hyclone, Cramlington, UK), 50 U/ml penicillin, 50 U/ml streptomycin (Hyclone) at 37°C under 5% CO_2_.

### RT-PCR

The presence or absence of retroviral mRNAs of four HERVs families (HERV-E, HERV-H, HERV-K and HERV-W), selected on the basis of those more frequently associated with human diseases, was assessed at T_0_ in PBMCs of ASDs and HCs by qualitative RT-PCR.

RNA isolation was performed using a NucleoSpin RNA kit according to the manufacturer's instructions (Machenery-Nagel, Dueren, Germany). Two hundred and fifty nanograms (250 ng) of DNase-treated RNA from ASD and HC PBMCs were reverse-transcribed into cDNA using the High Capacity cDNA Reverse Transcription Kit (Applied Biosystems, Life Technologies, Carlsbad CA) according to the manufacturer's protocol. Two hundred ng cDNA were amplified using specific primers for glyceraldehyde-3-phosphate dehydrogenase (GAPDH, forward, 5′-TGGTATCGTGAAAGGACT-3′; reverse, 5′-ATGCAAGTGAGCTTCCCGTTC-3′), as an internal control, or using degenerate primer pairs for HERV-E, HERV-H, HERV-K, HERV-W, to simultaneously evaluate the presence of different virus types belonging to an HERV family [26]. No RNA template control reactions were included in all experiments. The PCR products were visualized on 1.5% agarose gels containing 10 µg/ml ethidium bromide (EtBr) in 1× Tris-acetate-EDTA buffer. Samples in which PCR products could be visualized on EtBr-stained agarose gels were defined as positive for HERV family expression, while samples in which no specific band could be detected for any of the tested HERV families, yet positive for the GAPDH housekeeping gene, were defined as negative. All PCR products were sequenced to verify any false positives.

No template controls were included in all experiment.

### Real time PCR

The expression of the *env* of sequence from HERV-H and HERV-W families in PBMCs from both ASD and HC groups was quantitatively assessed in PBMC at T_0_ and T_72_, both in ASD and HC by Real-time quantitative PCR., The assays were performed in a Bio-Rad instrument (CFX96 Real-Time System), using SYBR Green chemistry (SYBR Real Green PCR Master Mix, Eppendorf). We selected specific pairs primers for *env* of HERV-H (Gene Bank accession number AJ289711; *env* forward, primer 5′ – TTCACTCCATCCTTGGCTAT – 3′; reverse, primer 5′ – CGTCGAGTATCTACGAGCAAT – 3′), for *env* of HERV-W (Gene Bank accession number NM_014590.3; forward, primer 5′ – CGTTCCATGTCCCCATTTTAG – 3′, reverse, primer 5′– TCATATCTAAGCCCCGCAAC – 3′) and for *env* of HERV-K (Gene Bank accession number AF1646; forward primer 5′ – CATGGCAATTCCCAGTAACTGT – 3′, reverse primer 5′ –CTCCCTCTTGGGCTCCTTCT – 3′). Each sample was analyzed in triplicate and a negative control, (no template reaction), was added included in each experiment, to check out any possible contamination. The house-keeping gene GUSB (Gene Bank accession number NM_000181; forward primer 5′- CAGTTCCCTCCAGCTTCAATG-3′; reverse, primer ACCCAGCCGACAAAATGC), was used to normalize the results. Each experiment was completed with a melting curve analysis to confirm the specificity of amplification and the lack of non-specific products and primer dimers. Quantification was performed using the threshold cycle (Ct) comparative method. The relative expression was calculated as follows: 2^−[ΔCt(sample) − ΔCt(calibrator)]^ = 2^−ΔΔCt^, where ΔCt (sample) = [Ct (HERV-H/W/K *env*) – Ct (GUSB)], and ΔCt (calibrator) was the mean of ΔCT of all of the controls at T_0_. Real time PCR results were represented by box plots, depicting mild (black dot) and extreme outliers (asterisk) for each group were showed.

### Statistical analysis

Fischer exact test was used to compare qualitative expression of HERV families. The Mann Whitney test was used to compare quantitative expression of HERVs families between ASD and HC groups at T_0_ or T_72_, and Wilcoxon test was used to compare stimulation response at T_72_ in each group. To determine any correlation between age and HERVs expression, the Spearman's rho correlation coefficient was calculated. The ANOVA analysis of variance and post-hoc Bonferroni tests were used to determine whether changes in the expression of HERV-H and HERV-W were associated with clinical parameters. Statistical analyses were done using the SPSS software (version 17.0). P values are indicated in the text, and in figures only for statistically significant comparisons (*p* values<0.050).

## Results

### Expression of HERV-H, W, K and E families in PBMCs from ASD patients and healthy controls

We first analysed the expression of four HERV families (H, W, K and E), selected on the basis of their frequent association with complex human diseases, in fresh (T_0_) and in culture stimulated (T_72_) PBMCs from both ASD and HC groups by qualitative RT-PCR. All amplification products were sequenced. Only two false positives were detected, which were excluded from this study. Table 3 reports the proportion, within the ASD and the HC groups, of positive individuals for specific HERV families, either selectively detected at T_0_, or selectively detected at T_72_, or detected at any one time (T_0_ or T_72_).

**Table 3:** Percentage of positive samples for expression of HERV families in cultured PBMCs from ASD and HC groups.

	*HERV-H*		*HERV-W*		*HERV-K*		*HERV-E*	
	*ASD*	*HC*	*ASD*	*HC*	*ASD*	*HC*	*ASD*	*HC*
***T_0_*** a	42.86 (12)	21.43 (6)	67.86 (19)	57.14 (16)	42.86 (12)	46.43 (13)	7.14 (2)	0 (0)
	*p* = 0.152		*p* = 0.582		*p* = 1.000		*p* = 0.491	
***T_72_*** b	44.44 (12)	25 (7)	81.48 (22)	60.71 (17)	55.56 (15)	46.43 (13)	0 (0)	0 (0)
	*p* = 0.162		*p* = 0.138		*p* = 0.593		*nc* d	
***T_0_ or T_72_*** c	71.43 (20)	35.71 (10)	89.29 (25)	67.86 (19)	64.29 (18)	71.43 (20)	7.14 (2)	0 (0)
	*p* = 0.015		*p* = 0.101		*p* = 0.775		*p* = 0.491	

a RT-PCR analysis of PBMCs at time 0 after withdrawal.

b RT-PCR analysis of PBMCs after 72 hours of stimulation in culture.

c RT-PCR analysis of PBMCs at T_0_ and T_72_.

d No statistics are computed.

In parentheses the number of positive samples over the total (28).

The percentage of HERV-H-expressing individuals was higher among ASD cases, compared to controls, at T_0_ (42.86% *vs* 21.43%), at T_72_ (44.44% *vs* 25%) and at least in one of the two times analysed (71.43% *vs* 35.71%). The differences between the ASD and HC groups, evaluated by Fisher exact test, are significant only when analyzing the percentage of positive individuals at T_0_ or T_72_ (*p* = 0.015), but not at T_0_ (*p* = 0.152) and at T_72_ (*p* = 0.162).

HERV-W was also more commonly detected among ASDs than in HCs, at both T_0_ (67.85% *vs* 57.14%) and T_72_ (81.48% *vs* 60.71%) and the percentage of positive samples at at least one of the analysed times was higher in ASDs (89.29%) compared to HCs (67.86%), albeit with no significant difference.

HERV-K was almost equally represented in both groups and in each of the assay conditions (at T_0_ ASD 42.86%, HC 46.43%; at T_72_ ASD 55.56%, HC 46.43%; at T_0_ or T_72_ ASD 64.29%, HC 71.43%) and no difference are detected by statistical analysis (ASD *vs* HC at T_0_ *p* = 1.000; at T_72_ *p* = 0.593; at T_0_ or T_72_ *p* = 0.775). HERV-E was poorly expressed in ASD patients (7.14%) and absent in HCs, at both times of the analysis.

### Analysis of HERV-H, HERV-W and HERV-K expression in PBMCs of ASD patients and healthy controls

We next assessed the expression levels of *env* sequence from HERV-H, W and K families (but not HERV-E, due to its low representation in qualitative RT-PCR assays) in PBMCs from both ASD and HC groups. Real time assays were performed immediately after collection (T_0_) and after *in vitro* stimulation (T_72_), with the intent to investigate HERVs expression in resting *versus* proliferating conditions.

**Figure 1:**
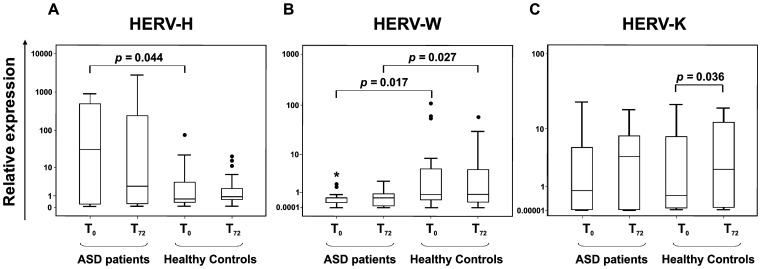
Relative HERV-H, W and K expression in ASD patients and healthy controls. Data obtained in fresh PBMCs (T_0_) and after *in vitro* stimulation (T_72_) of ASD patients and healthy controls are represented as box plot. ASD patients show higher levels of HERV-H (panel A) both at T_0_ and after *in vitro* stimulation in comparison to healthy controls. Conversely, HERV-W (panel B) expression is higher in healthy controls than in ASD patients, both at T_0_ and after *in vitro* stimulation. Significant differences between groups are shown. HERV-K (panel C) expression levels were similar in ASDs compared to HCs in both conditions, and significant differences were achieved only inside control group. Relative env gene expression levels were analyzed by Real-time PCR and represented by 2^−ΔΔCt^ in logarithmic scale.

At T_0_, HERV-H expression (Figure 1, panel A), shown as log (2^−ΔΔCt^), was significantly more elevated in fresh PBMCs from ASDs (median value 86.35; Interquartile range, IQR = 0.20/498.39) compared to the expression level in the HC group (median value 0.65; IQR = 0.35/4.48) (*p* = 0.044). After *in vitro* stimulation (T_72_), HERV-H expression (Figure 1, panel A) was still more elevated in ASD (median value 2.55; IQR = 0.25/258.11) than in HC group (median value 0.89; IQR = 0.57/2.34), though the statistical differences were not significant. In contrast, HERV-W was significantly more expressed in PBMCs from HCs compared to ASD patients (Figure 1, panel B), both at T_0_ and T_72_. In particular, the median value at T_0_ was 0.56 (IQR = 0.25/0.57) for ASDs, while being 0.80 (IQR = 0.38/5.15) (*p* = 0.017) for HCs. At T_72_ the median value was 0.56 (IQR = 0.09/0.9) for ASDs and 0.82 (IQR = 0.28/5.00) (*p* = 0.027) for HCs. Finally, HERV-K expression levels were similar in ASDs compared to HCs, at both analysed times (Figure 1, panel C). In fresh PBMCs from ASDs median value was 1.06 (IQR = 0.021/5.37) compared to 0.54 in HCs (IQR = 0.06/7.87) (*p* = 0.077), and after *in vitro* stimulation it rose to 3.88 (IQR = 0.01/7.90) in ASDs *versus* 2.35 (IQR = 0.09/12.53) in HCs (*p* = 0.694). Significant differences in HERV-K expression levels between T_0_ and T_72_ were achieved only in HC group (*p* = 0.036), but not between patients and controls.

**Figure 2:**
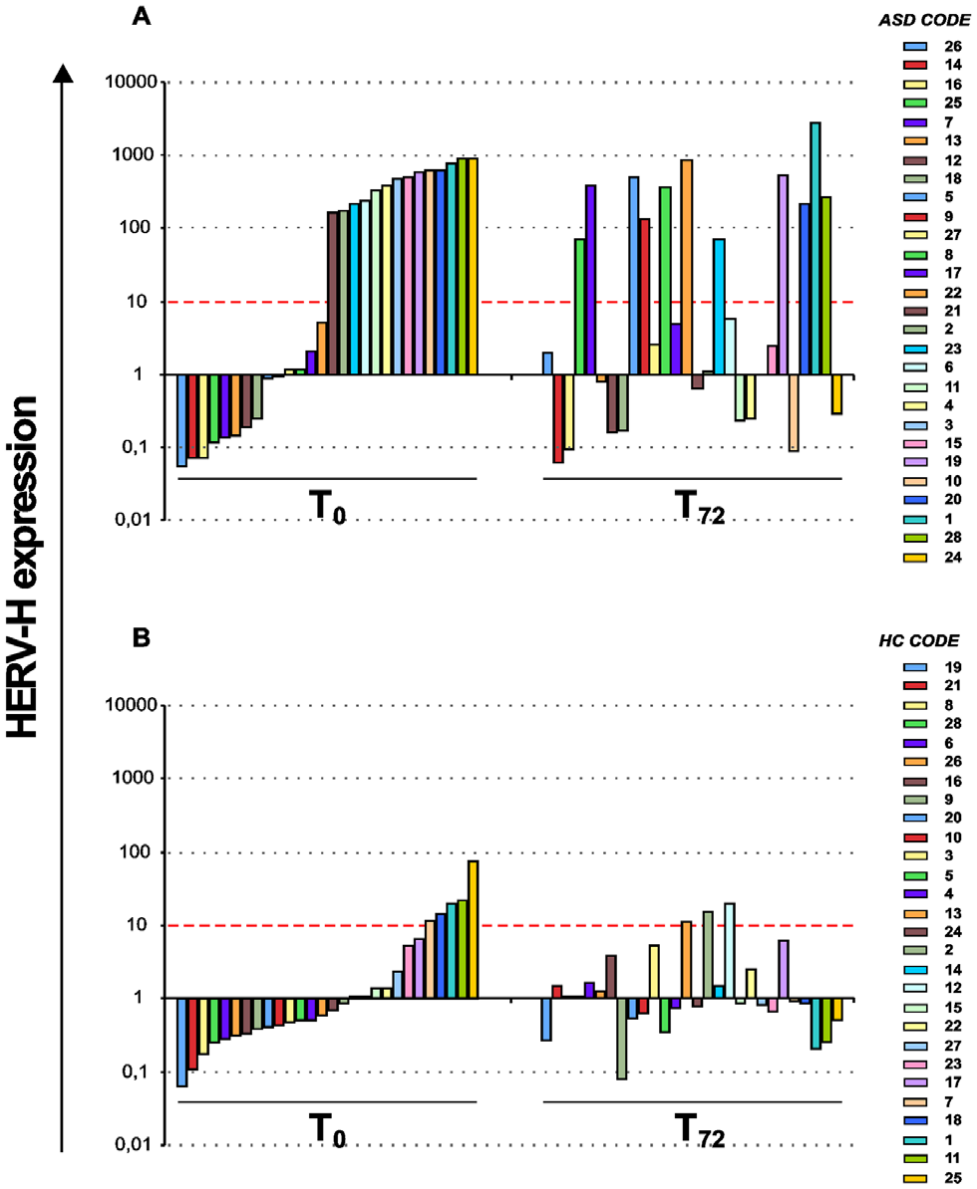
Quantitative evaluation of HERV-H expression in individual ASD patients and healthy controls. Comparative analysis of expression levels in individual ASD patients (panel A) shows that *in vitro* stimulation of PBMCs induces HERV-H expression increase in the patients with low levels at T_0_. Conversely, *in vitro* stimulation does not modify HERV-H levels in individual HC (panel B). Results obtained by Real time PCR are represented as 2^−ΔΔCT^ in logarithmic scale. Red dashed line identifies values 2^−ΔΔCT^>10.

The individual quantitative evaluation of HERV-H expression showed that 50% of ASD patients (14/28) exhibited very high levels (2^−ΔΔCT^>10) at T_0_; in addition, six of the samples showing low levels at T_0_ (2^−ΔΔCT^<10) up-regulated HERV-H levels after stimulation in culture. As a result, the overall frequency of HERV-H highly expressing ASD patients was higher than 70% (20/28) (Figure 2A). HERV-H expression was instead generally lower in the HC group: high levels (2^−ΔΔCT^>10) were detected in only a few individuals at T_0_ (5/28) and, among low-expressing healthy controls at T_0_ (23/28), only 3 showed increased expression after *in vitro* stimulation (Figure 2B).

**Figure 3:**
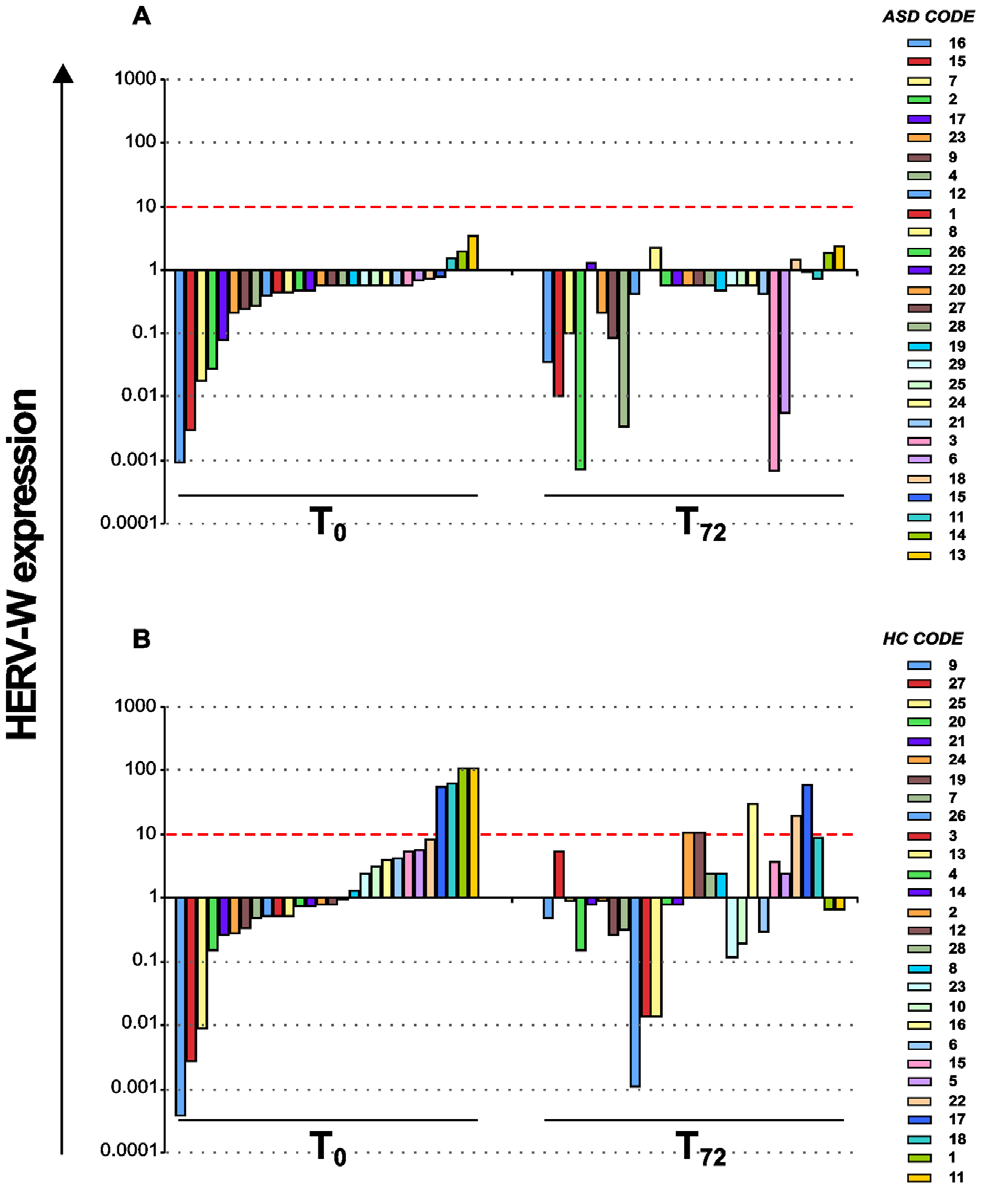
Quantitative evaluation of HERV-W expression in individual ASD patients and healthy controls. No significant differences in expression levels of individual ASD patients (panel A) and healthy control (panel B) after *in vitro* stimulation are observed. Results obtained by Real time PCR are represented as 2^−ΔΔCT^>10 in logarithmic scale. Red dashed line identifies values 2^−ΔΔCT^>10.

Finally, the individual expression analysis of HERV-W elements showed that the *in vitro* stimulation did not significantly modify their expression levels, in either the ASD or in the HC group (Figure 3A and 3B).

In summary, therefore, the results pinpoint two distinctive features of HERV elements in ASD: i.e. a significant overexpression of HERV-H, paralleled by a significant down-regulation of HERV-W in PBMCs from ASD patients compared to controls. Moreover the analysis of individual patients and controls highlighted an intrinsic potential of PBMCs from ASD patients to express HERV-H after stimulation in culture, unlike healthy controls.

### Correlation analysis of HERV-H and HERV-W expression with age

**Figure 4:**
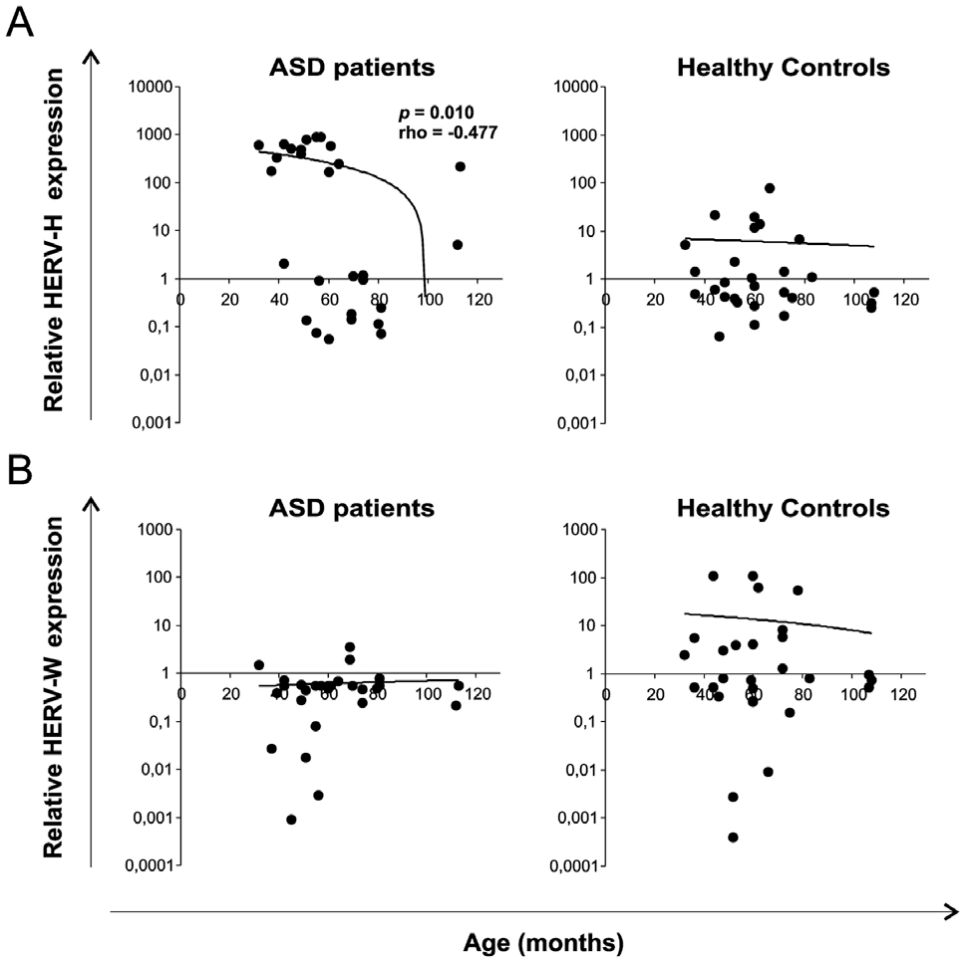
Association of HERV-H and W expression levels with the age of ASD patients and healthy controls. HERV-H (panel A) and HERV-W (panel B) levels at T_0_ are plotted as a function of corresponding age expressed in months. Spearman analysis shows a significant negative correlation between HERV-H expression levels and age only in ASD patients (rho = −0.477; *p* = 0.010). No significant correlation was found between HERV-W expression and age of both ASD and HC subjects. Relative env gene expression levels were analyzed by Real-time PCR and represented by 2^−ΔΔCt^ in logarithmic scale.

We next performed a Spearman correlation analysis between the expression of HERV-H and HERV-W, both of which showed a distinctive modulation in the ASD group, with age. Figure 4 shows the HERV-H and HERV-W env gene expression levels, evaluated by Real time PCR analysis, in PBMCs, plotted against age, expressed in months. Statistical analysis demonstrated a significant negative correlation between the expression of HERV-H at T_0_ and age in ASD patients (rho = −0.477; *p* = 0.010) but not in HCs (rho = −0.117; *p* = 0.553) (Figure 4, panel A). No significant correlation emerged between the expression of HERV-W and age either in the ASD or in the HC group (ASDs: rho = 0.145, *p* = 0.460; HCs: rho = 0.013, *p* = 0.948) (Figure 4, panel B). Thus, the correlation analysis suggests that higher levels of HERV-H are associated to lower age of the ASD patients.

### High levels of HERV-H expression in PBMCs of autistic patients with severe score in Communication and Motor Psychoeducational Profile-3

We next evaluated the association of HERV-H and HERV-W *env* expression (mean values) in fresh PBMCs with the PEP-3 classification (Psycho-educational Profile-Third edition).

**Figure 5:**
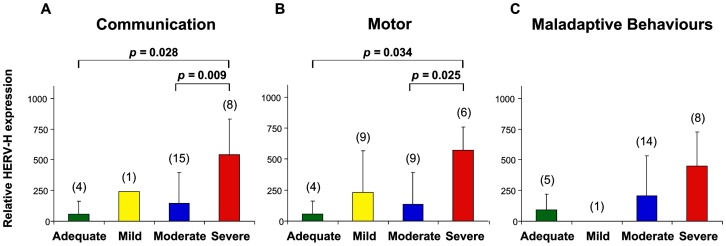
HERV-H expression and Psychoeducational Profile-3 in ASD patients. The skills and behaviours of individual cases were classified as Adequate, Mild, Moderate or Severe according to the degree of impairment for Communication PEP-3 (panel A), Motor PEP-3 (panel B) and Maladaptive Behavior PEP-3 (panel C) analysis. Mean values ± SD of HERV-H *env* expression in PBMCs at T_0_ from 28 ASD patients were analyzed. In parenthesis the number of patients classified for each analysed domain. Significant differences between groups are shown. Relative env gene expression levels were analyzed by Real-time PCR and represented by 2^−ΔΔCt^ in linear scale.

Figure 5 shows the mean values ± SD of HERV-H expression in PBMCs at T_0_ from the 28 ASD patients, grouped in four developmental levels (Adequate, Mild, Moderate and Severe) and resulted by analysis of the three areas of analysis Communication (panel A), Motor (panel B) and Maladaptive Behaviours (panel C).

The relative HERV-H expression mean values were found to be significantly higher in ASD patients with Severe impairment level in Communication compared with those with Moderate or Adequate level (mean values ± SD: Adequate 55.49±106.20, Mild 106.20, Moderate 143.26±254.81, Severe 539.62±292.78; Severe *vs* Adequate *p* = 0.028, Severe *vs* Moderate *p* = 0.009) (Figure 5, panel A). When the PEP-3 Motor analysis was considered, the relative HERV-H expression mean values were again found to be significantly higher in ASD patients with Severe compared with those with Moderate or Adequate level (mean values ± SD: Adequate 55.49±106.20, Mild 231.76±335.04, Moderate 133.78±259.37, Severe 570.01±187.54; Severe *vs* Adequate *p* = 0.034, Severe *vs* Moderate *p* = 0.025) (Figure 5, panel B). Finally, for the PEP-3 analysis of Maladaptive Behaviours, HERV-H expression mean values were higher in ASD patients with Severe impairment level though with no significant differences (mean values ± SD: Adequate 93.20±124.77, Mild 0.07, Moderate 204.87±328.41, Severe 449.70±277.47) (Figure 5, panel C). No statistically significant differences were found in HERV-W *env* expression when ASD patients were stratified for the four developmental levels defined by the PEP-3 analysis (data not shown).

Thus, high levels of HERV-H expression are associated to ASD patients with Severe impairment of developmental level, as defined by the Communication and Motor analysis.

## Discussion

Despite extensive research efforts, the etiopathogenesis of ASD thus far remains elusive. To date ASD remains a behaviorally defined spectrum with no known biological markers suitable to support diagnosis or subgroup categorization [27].

The genetic architecture of ASD is highly heterogeneous [28], and only about 10–20% of individuals with ASD have an identified genetic etiology [29]. The transmission pattern is complex in most families and is not compatible with simple Mendelian inheritance [30], [31], suggesting that protein-coding genes are responsible for only part of the ASD etiology. Growing evidence supports the involvement of epigenetic regulatory mechanisms in the pathogenesis of ASD [32], [33], with a contribution of DNA methylation, genomic imprinting, chromatin modifications and non coding RNA [34].

As for other complex diseases, the full etiology most probably relies on a complex interplay between genes, the genome organization and the environment. A well-understood example of such interplay comes from studies of the Rett syndrome. It is worth recalling that LINE-1 elements, a retrotransposon family accounting for 17% of the human genome, play fundamental roles in neurogenesis by altering the expression of neuronal genes, which, in turn, influence neuronal cell fate [35]. Rett syndrome patients have recently been found to display an increased susceptibility to LINE-1 retrotransposition, dependent on the abnormal methylation status of the overall genome due to mutation of the DNA methyl-binding protein, MeCP2 [36], which is regarded as the causative alteration of the Rett disease.

Importantly, growing evidence links germline hypomethylation and genomic instability. Structural mutations in individuals with schizophrenia, bipolar disorder, developmental retardation and autism are significantly more concentrated within hypomethylated regions, suggesting a connection between the methylation status of genomic DNA and human disease [37].

Here we have tested the hypothesis that HERVs - a component of human mobile retrotransposon families [38] - play roles in the onset or progression of the disease. Based on their ability to be mobilized under specific stimuli, HERVs might actually be considered as emerging pathogens and can be seen as spanning the bridge between genetic predisposition and environmental factors. Their responsiveness to environmental conditions is an intrinsic property that places them at the frontline of the gene-environment interaction. HERVs are also formidable evolutionary forces that have shaped the architecture of the genomes of higher organisms, with some conserving the ability to induce new integrants within their host's genome [17]. Recent studies have disclosed unsuspected effects of retroelements in genome-wide modulation of the transcriptome [39] in fundamental processes such as embryogenesis [17] and in a variety of pathologies [40], including complex brain disorders, eg, schizophrenia [23]–[25].

We have studied four HERV families in PBMCs in an attempt to identify molecular signatures of ASD that may be easily detected in peripheral samples. An increasing number of molecular studies indeed indicate the importance of differential expression of ASD-associated genes in peripheral tissues, as well as in postmortem brains, from ASD subjects [41]–[43]. In particular, monozygotic twins, discordant for diagnosis of autism, were reported to show differential gene expression in lymphoblastoid cell lines [41].

The data presented here indicate that the percentage of HERV-H and HERV-W positive samples, evaluated in fresh and in culture stimulated PBMCs by qualitative RT-PCR, is higher in cases compared to controls, while HERV-K shows only minimal differences and HERV-E was virtually absent. When considering all positive samples in either condition (fresh or stimulated), the differences were significant for HERV-H, but not for the other HERV families analyzed. Quantitative determination of HERVs in PBMCs showed that HERV-H expression, indeed, was statistically significantly higher in ASD, and, conversely, HERV-W was higher in healthy controls.

Furthermore, HERV-H expression negatively correlated with age only in ASD patients. Based on the evidence that HERV-H is expressed in high levels selectively in ASDs, the correlation with age might be viewed as a disease-dependent feature not present in HCs.

Interestingly, high expression of HERV-H was also associated with “severe” score in Communication and Motor Psychoeducational Profile-3.

To the best of our knowledge, this is the first evidence linking retrotransposon activity and ASD. Notwithstanding the relatively small size of the samples tested in this work, the statistical significance of the present findings supports the hypothesis that HERV-H overexpression might be regarded as a potential early marker detectable in ASD patients. The analysis of individual patients and controls also suggests an increased intrinsic predisposition of the PBMCs from ASD patients to express HERV-H in response to mitogenic stimulation in culture. HERV-H overexpression might be of help to differentiate young ASD children from age-matched controls. Because detecting autism at the earliest possible age is of outmost importance to optimize outcomes for children with the disorder, identifying the presence of HERV-H in PBMCs of young children could be useful for this purpose. Furthermore, because autism remains a behaviorally defined disorder, the identification of a biological marker could also be of support for a confident diagnosis. The identification of a reliable biomarker for ASD could supplement and validate existing clinical methods; in particular, a biomarker that is expressed at, or even before, the onset of symptoms might obviate the need to wait for behavioral criteria to be met before beginning treatment.

Larger number of ASD patients and follow-up data will be needed to further substantiate the present results. Yet, as the first comparative analysis of ASD patients and controls focusing on HERV families, we believe that the present findings are well worth pursuing in future research. More generally, the quantitative differences in HERV-H and HERV-W *env* expression between ASD patients and controls suggest a contribution of a “non-coding” fraction of the genome to ASD.

## Conclusions

Our results demonstrate that the expression of two particular HERV families is distinctive of ASD and may represent a possible molecular marker for ASD patients. HERVs may be thought of as components of the genome that interact with environmental factors and/or infectious agents, potentially capable to interplay with different molecular pathways in determining individual genetic differences in ASD.

## References

[1] American Psychiatric Association. Diagnostic and statistical manual of mental disorder 4*^th^* edn, (2004) Washington DC: American Psychiatric Press.

[2] Aldinger KA, Plummer JT, Qiu S, Levitt P (2011) SnapShot: genetics of Autism. Neuron 72: 418–8.e1. 22017998 10.1016/j.neuron.2011.10.007

[3] Benvenuto A, Moavero R, Alessandrelli R, Manzi B, Curatolo P (2009) Syndromic autism: causes and pathogenetic pathways. World J Pediatr 5: 169–176. 19693459 10.1007/s12519-009-0033-2

[4] Lupski JR, Belmont JW, Boerwinkle E, Gibbs RA (2011) Clan genomics and the complex architecture of human disease. Cell 147: 32–43. 21962505 10.1016/j.cell.2011.09.008 PMC3656718

[5] Baillie JK, Barnett MW, Upton KR, Gerhardt DJ, Richmond TA, et al (2011) Somatic retrotransposition alters the genetic landscape of human brain. Nature 10.1038/nature10531. 10.1038/nature10531 PMC3224101 22037309

[6] Ben-David E, Granot-Hershkovitz E, Monderer-Rothkoff G, Lerer E, Levi S, et al (2011) Identification of a functional rare variant in autism using genome-wide screen for monoallelic expression. Hum Mol Genet 20: 3632–3641. 21680558 10.1093/hmg/ddr283

[7] Salyakina D, Cukier HN, Lee JM, Sacharow S, Nations LD, et al (2011) Copy number variants in extended autism spectrum disorder families reveal candidates potentially involved in autism risk. PLoS One 6: e26049. 22016809 10.1371/journal.pone.0026049 PMC3189231

[8] Günther T, Schmitt AO, Bortfeldt RH, Hinney A, Hebebrand J, et al (2011) Where in the genome are significant nucleotide polymorphisms from genome-wide association studies located? OMICS 15: 507–512. 21699402 10.1089/omi.2010.0154

[9] International Human Genome Consortium (2001) Initial sequencing and analysis of the human genome. Nature 409: 860–921. 11237011 10.1038/35057062

[10] Huang CR, Schneider AM, Lu Y, Niranjan T, Shen P, et al (2010) Mobile interspersed repeats are major structural variants in the human genome. Cell 141: 1171–1182. 20602999 10.1016/j.cell.2010.05.026 PMC2943426

[11] Iskow RC, McCabe MT, Mills RE, Torene S, Pittard WS, et al (2010) Natural mutagenesis of human genomes by endogenous retrotransposons. Cell 141: 1253–1261. 20603005 10.1016/j.cell.2010.05.020 PMC2943760

[12] Boeke JD, Stoye JP (1997) Retrotrasposons, endogenous retroviruses, and the evolution of retroelements. In: Retroviruses, Coffin JM, Hughes SH and Varmus HE editors. New York: Cold Spring Harbor Laboratory Press. pp. 343–346. 21433351

[13] Forsman A, Yun Z, Hu L, Uzhameckis D, Jern P, et al (2005) Development of broadly targeted human endogenous gammaretroviral pol-based real time PCRs Quantitation of RNA expression in human tissues. J Virol Methods 129: 16–30. 15967513 10.1016/j.jviromet.2005.04.016

[14] Seifarth W, Frank O, Zeilfelder U, Spiess B, Greenwood AD, et al (2005) Comprehensive analysis of human endogenous retrovirus transcriptional activity in human tissues with a retrovirus-specific microarray. J Virol 79: 341–352. 15596828 10.1128/JVI.79.1.341-352.2005 PMC538696

[15] Yi JM, Kim HM, Kim HS (2006) Human endogenous retrovirus HERV-H family in human tissues and cancer cells: expression, identification, and phylogeny. Cancer Lett 231: 228–239. 16399224 10.1016/j.canlet.2005.02.001

[16] Bannert N, Kurth R (2006) The evolutionary dynamics of human endogenous retroviral families. Annu Rev Genomics Hum Genet 7: 149–173. 16722807 10.1146/annurev.genom.7.080505.115700

[17] Rowe HM, Trono D (2011) Dynamic control of endogenous retroviruses during development. Virology 411: 273–287. 21251689 10.1016/j.virol.2010.12.007

[18] Marguerat S, Wang WY, Todd JA, Conrad B (2004) Association of human endogenous retrovirus K-18 polymorphisms with type 1 diabetes. Diabetes 53: 852–854. 14988274 10.2337/diabetes.53.3.852

[19] Ryan FP (2009) An alternative approach to medical genetics based on modern evolutionary biology. Part 4: HERVs in cancer. J R Soc Med 102: 474–480. 19875536 10.1258/jrsm.2009.090289 PMC2770355

[20] Serafino A, Balestrieri E, Pierimarchi P, Matteucci C, Moroni G, et al (2009) The activation of human endogenous retrovirus K (HERV-K) is implicated in melanoma cell malignant transformation. Exp Cell Res 315: 849–862. 19167380 10.1016/j.yexcr.2008.12.023

[21] Balada E, Ordi-Ros J, Vilardell-Tarrés M (2009) Molecular mechanisms mediated by human endogenous retroviruses (HERVs) in autoimmunity. Rev Med Virol 19: 273–286. 19714703 10.1002/rmv.622

[22] Christensen T (2005) Association of human endogenous retroviruses with multiple sclerosis and possible interactions with herpes viruses. Rev Med Virol 15: 179–211. 15782388 10.1002/rmv.465

[23] Yao Y, Schröder J, Nellaker C, Bottmer C, Bachmann S, et al (2008) Elevated levels of human endogenous retrovirus-W transcripts in blood cells from patients with first episode schizophrenia. Genes Brain Behav 7: 103–112. 17559415 10.1111/j.1601-183X.2007.00334.x

[24] Weis S, Llenos IC, Sabunciyan S, Dulay JR, Isler L, et al (2007) Reduced expression of human endogenous retrovirus (HERV)-W GAG protein in the cingulate gyrus and hippocampus in schizophrenia, bipolar disorder, and depression. J Neural Transm 114: 645–655. 17219017 10.1007/s00702-006-0599-y

[25] Frank O, Giehl M, Zheng C, Hehlmann R, Leib-Mösch C, et al (2005) Human endogenous retrovirus expression profiles in samples from brains of patients with schizophrenia and bipolar disorders. J Virol 79: 10890–10901. 16103141 10.1128/JVI.79.17.10890-10901.2005 PMC1193590

[26] Johnston JB, Silva C, Holden J, Warren KG, Clark AW, et al (2001) Monocyte activation and differentiation augment human endogenous retrovirus expression: implications for inflammatory brain diseases. Ann Neurol 50: 434–442. 11601494 10.1002/ana.1131

[27] Anagnostou E, Taylor MJ (2011) Review of neuroimaging in autism spectrum disorders: what have we learned and where we go from here. Mol Autism 2: 4. 21501488 10.1186/2040-2392-2-4 PMC3102613

[28] Abrahams BS, Geschwind DH (2008) Advances in autism genetics: on the threshold of a new neurobiology. Nat Rev Genet 9: 341–355. 18414403 10.1038/nrg2346 PMC2756414

[29] Bill BR, Geschwind DH (2009) Genetic advances in autism: heterogeneity and convergence on shared pathways. Curr Opin Genet Dev 19: 271–278. 19477629 10.1016/j.gde.2009.04.004 PMC2715429

[30] Jorde LB, Hasstedt SJ, Ritvo ER, Mason-Brothers A, Freeman BJ, et al (1991) Complex segregation analysis of autism. Am J Hum Genet 49: 932–938. 1928098 PMC1683259

[31] Autism Genome Project Consortium (2007) Mapping autism risk loci using genetic linkage and chromosomal rearrangements. Nat Genet 39: 319–328. 17322880 10.1038/ng1985 PMC4867008

[32] Schanen NC (2006) Epigenetics of autism spectrum disorders. Hum Mol Genet 15 Spec No 2: R138–150. 16987877 10.1093/hmg/ddl213

[33] Beaudet AL (2007) Autism: highly heritable but not inherited. Nat Med 13: 534–536. 17479094 10.1038/nm0507-534

[34] LaSalle JM (2011) A genomic point-of-view on environmental factors influencing the human brain methylome. Epigenetics 6: 862–869. 21617367 10.4161/epi.6.7.16353 PMC3154427

[35] Muotri AR, Chu VT, Marchetto MC, Deng W, Moran JV, et al (2005) Somatic mosaicism in neuronal precursor cells mediated by L1 retrotransposition. Nature 435: 903–910. 15959507 10.1038/nature03663

[36] Muotri AR, Marchetto MC, Coufal NG, Oefner R, Yeo G, et al (2010) L1 retrotransposition in neurons is modulated by MeCP2. Nature 468: 443–446. 21085180 10.1038/nature09544 PMC3059197

[37] Li J, Harris RA, Cheung SW, Coarfa C, Jeong M, et al (2012) Genomic hypometilation in the human germline associates with selective structural mutability in the human genome. Plos genetics 8 (5) 10.1371/journal.pgen.1002692 PMC3355074 22615578

[38] Kurth R, Bannert N (2010) Beneficial and detrimental effects of human endogenous retroviruses. Int J Cancer 126: 306–314. 19795446 10.1002/ijc.24902

[39] Faulkner GJ, Kimura Y, Daub CO, Wani S, Plessy C, et al (2009) The regulated retrotransposon transcriptome of mammalian cells. Nat Genet 41: 563–571. 19377475 10.1038/ng.368

[40] Perron H, Lang A (2010) The human endogenous retrovirus link between genes and environment in multiple sclerosis and in multifactorial diseases associating neuroinflammation. Clin Rev Allergy Immunol 39: 51–61. 19697163 10.1007/s12016-009-8170-x

[41] Hu VW, Frank BC, Heine S, Lee NH, Quackenbush J (2006) Gene expression profiling of lymphoblastoid cell lines from monozygotic twins discordant in severity of autism reveals differential regulation of neurologically relevant genes. BMC Genomics 7: 118. 16709250 10.1186/1471-2164-7-118 PMC1525191

[42] Baron CA, Liu SY, Hicks C, Gregg JP (2006) Utilization of lymphoblastoid cell lines as a system for the molecular modeling of autism. J Autism Dev Disord 36: 973–982. 16845580 10.1007/s10803-006-0134-x

[43] Nishimura Y, Martin CL, Vazquez-Lopez A, Spence SJ, Alvarez-Retuerto AI, et al (2007) Genome-wide expression profiling of lymphoblastoid cell lines distinguishes different forms of autism and reveals shared pathways. Hum Mol Genet 16: 1682–1698. 17519220 10.1093/hmg/ddm116

